# Functional Analysis of Variants in Complement Factor I Identified in Age-Related Macular Degeneration and Atypical Hemolytic Uremic Syndrome

**DOI:** 10.3389/fimmu.2021.789897

**Published:** 2022-01-05

**Authors:** Sarah de Jong, Anita de Breuk, Bjorn Bakker, Suresh Katti, Carel B. Hoyng, Sara C. Nilsson, Anna M. Blom, Lambert P. van den Heuvel, Anneke I. den Hollander, Elena B. Volokhina

**Affiliations:** ^1^ Department of Ophthalmology, Donders Institute for Brain, Cognition and Behavior, Radboud University Medical Center, Nijmegen, Netherlands; ^2^ Gemini Therapeutics Inc., Cambridge, MA, United States; ^3^ Department of Translational Medicine, Lund University, Malmö, Sweden; ^4^ Amalia Children’s Hospital, Radboud University Medical Center, Nijmegen, Netherlands; ^5^ Radboud Institute for Molecular Life Sciences, Radboud University Medical Center, Nijmegen, Netherlands; ^6^ Department of Laboratory Medicine, Radboud University Medical Center, Nijmegen, Netherlands; ^7^ Department of Human Genetics, Radboud University Medical Center, Nijmegen, Netherlands

**Keywords:** complement factor I, genetic variants, age-related macular degeneration, atypical hemolytic uremic syndrome, functional analysis

## Abstract

Complement factor I (FI) is a central inhibitor of the complement system, and impaired FI function increases complement activation, contributing to diseases such as age-related macular degeneration (AMD) and atypical hemolytic uremic syndrome (aHUS). Genetic variation in complement factor I (*CFI*) has been identified in both AMD and aHUS, with more than half of these variants leading to reduced FI secretion levels. For many of the variants with normal FI secretion, however, functional implications are not yet known. Here we studied 11 rare missense variants, with FI secretion levels comparable to wildtype, but a predicted damaging effects based on the Combined Annotation Dependent Depletion (CADD) score. Three variants (p.Pro50Ala, p.Arg339Gln, and p.Ser570Thr) were analyzed in plasma and serum samples of carriers affected by AMD. All 11 variants (nine for the first time in this study) were recombinantly expressed and the ability to degrade C3b was studied with the C3b degradation assay. The amount of degradation was determined by measuring the degradation product iC3b with ELISA. Eight of 11 (73%) mutant proteins (p.Pro50Ala, p.Arg339Gln, p.Ile340Thr, p.Gly342Glu, p.Gly349Arg, p.Arg474Gln, p.Gly487Cys, and p.Gly512Ser) showed significantly impaired C3b degradation, and were therefore classified as likely pathogenic. Our data indicate that genetic variants in *CFI* with a CADD score >20 are likely to affect FI function, and that monitoring iC3b in a degradation assay is a useful tool to establish the pathogenicity of *CFI* variants in functional studies.

## Introduction

The complement system is a part of innate immunity, playing a crucial role in the first line of defense against pathogens. Complement can be activated *via* three different pathways: the alternative pathway, the classical pathway or the lectin pathway. The alternative pathway is spontaneously activated at a low-level by tick-over of C3 to C3(H_2_O), which resembles the activation fragment C3b. Both C3(H_2_O) and C3b recruit factor B (FB), which is then cleaved by factor D, resulting in release of the fragment Ba and formation of the C3 convertase C3(H_2_O)Bb or C3bBb. C3bBb is bound and stabilized by the complement factor Properdin (FP), forming C3bBbP. The C3 convertase cleaves C3 into C3b and the anaphylatoxin C3a, initiating an amplification loop. Additional, binding of C3b to the C3bBbP complex generates the C5 convertase C3bBbPC3b, which cleaves C5 into C5a and C5b. C5b initiates formation of the membrane attack complex by sequential binding of C6, C7, C8 and multiple C9 molecules. In light of continuous activation and potential for rapid amplification of the alternative pathway, it is not surprising that inhibitors of complement activation play a crucial role in protecting host tissue. Factor I (FI) is a central inhibitor of complement activation and prevents excessive complement activation by degrading C3b and C4b, the activation product of the lectin and classical pathways, in the presence of a co-factor. One of these co-factors is Factor H (FH), which facilitates C3b degradation only (1).

Genetic variation in complement factor I (*CFI*) leading to reduced FI levels or impaired FI function has been associated with diseases such as age-related macular degeneration (AMD) and atypical hemolytic uremic syndrome (aHUS) (2, 3). AMD is a common disease among the elderly, leading to vision loss in advanced stages. It is hallmarked by accumulation of extracellular debris between the retinal pigment epithelium (RPE) and Bruch’s membrane. Drusen expansion and fusion lead to degeneration of the RPE, followed by photoreceptor death (geographic atrophy), or the formation of new blood vessels invading the retina (neovascular AMD). These blood vessels tend to break and leak, leading to rapid vision loss (4). aHUS, on the other side, is a rare kidney disorder, hallmarked by microangiopathic thrombocytopenia, hemolytic anemia and acute renal failure (5).

In AMD patients a younger age of onset was reported in carriers of rare variants in *CFI* (6), and in aHUS patients chances of recurrence were high after kidney transplantation in carriers of pathogenic *CFI* variants (7). In order to determine the pathogenicity of rare coding variants in *CFI* we previously performed a comprehensive analysis of protein secretion levels for 126 rare coding variants identified in patients with AMD, aHUS or C3-glomerulopathy. Of the tested variants 68 (54%) showed significantly reduced FI secretion levels *in vitro* (8). However, also variants with normal FI secretion can potentially have impaired functional activity.

**Figure 1:**
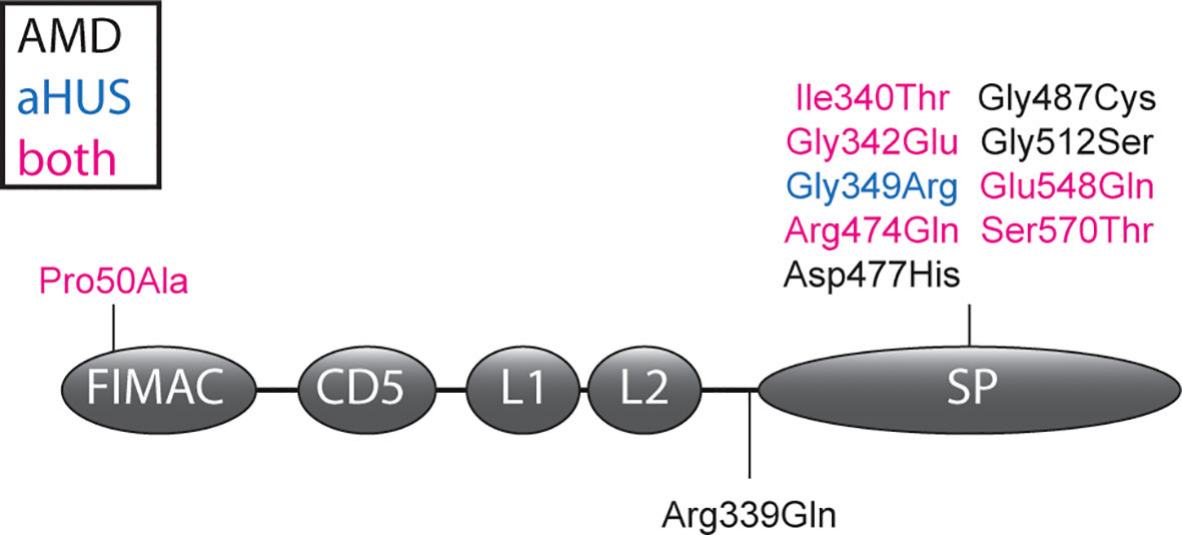
*CFI* variants analyzed in this study. The amino acid changes due to *CFI* variants are shown in the FI domain scheme. Variants are colored depending whether they were identified in AMD patients (black), aHUS patients (blue) or both in AMD and aHUS patients (magenta). Details of the shown variants are summarized in **Supplementary Table 1**. FIMAC, Factor I membrane attack complex domain; L1 and L2, low-density lipoprotein receptor 1 and 2 domains; SP, serine proteinase domain.

Therefore in this study, we aimed to perform functional analysis for 11 variants that are predicted to be damaging based on the Combined Annotation Dependent Depletion (CADD) score, but exhibited FI secretion comparable to WT (**Figure 1**). Functionality of FI was evaluated with a C3b degradation assay followed by measurement of the degradation product iC3b with ELISA.

## Materials and Methods

### Study Population

Carriers of a rare variant in *CFI* with a CADD score >20 and reported normal FI secretion levels (8) were selected from the European Genetic Database (EUGENDA). All enrolled participants provided written informed consent to participate in the study and were pseudo-anonymized with a database identifier code. The study was approved by the local ethics committee region Arnhem/Nijmegen on Research Involving Human Subjects and conducted according to the Declaration of Helsinki.

In total seven carriers of a rare *CFI* variant not affecting FI secretion were included. In addition two carriers of the pathogenic c.355G>A (p.Gly119Arg) variant (8–13), and 17 individuals not carrying a rare or low-frequency variant in *CFI*, *CFH*, *CFB* or *C3* (**Table 1**) were included in the control group.

**Table 1:** *CFI* rare variant carriers included in this study.

Individual	cDNA change	Protein change	plasma/serum available	Phenotype at sampling time point	Age (years) at sampling time point	Gender	Age at diagnosis	Family history^a^	Progression to a more severe phenotype at later visits
1	355G>A	Gly119Arg	yes	Advanced AMD: neovascular AMD	69.9	female	60.20	Yes	No
2	355G>A	Gly119Arg	yes	early AMD	75.8	male	75.80	No	Yes
3	148C>G	Pro50Ala	yes	intermediate AMD	78.0	female	78.04	No	Yes
4	148C>G	Pro50Ala	yes	intermediate AMD	45.2	male	42.93	Yes	No
5	1016G>A	Arg339Gln	yes	no AMD	56.6	male	n/a	Yes	Unknown^b^
6	1016G>A	Arg339Gln	yes	early AMD	54.1	male	54.05	Yes	No
7	1709G>C	Ser570Thr	yes	Advanced AMD: neovascular AMD	74.5	female	74.27	No	Unknown^b^
8	1709G>C	Ser570Thr	yes	early AMD	79.4	female	79.35	No	No
9	1709G>C	Ser570Thr	only plasma	no AMD	49.1	male	n/a	Yes	Unknown^b^

a based on self-reported questionnaires.

b no follow-up information available.

AMD diagnosis was obtained by evaluation of retinal images according to the Cologne Image Reading Center and Laboratory (CIRCL) protocol in 17 individuals (14). In 9 individuals CIRCL grading was not available, and retinal images were graded according to the Wisconsin age-related maculopathy grading system (WARMGS) and reclassified into the Rotterdam Classification (RC) (15–17). All individuals were assigned to one of the four phenotype groups: no AMD (no signs of AMD or ≤10 small drusen ≤63 μm together with pigmentary changes), early AMD (>10 small drusen ≤63 μm or presence of intermediate drusen 63-124 μm), intermediate AMD (large drusen ≥125 µm diameter or ≥15 intermediate drusen 63-124 μm), or advanced AMD (choroidal neovascularization or geographic atrophy in at least one eye).

EDTA plasma and serum samples were collected and centrifuged according to standard protocols and frozen at −80^◦^C within 1 h. The samples were stored at −80^◦^C at the Ophthalmology department of the Radboud university medical center or in the Radboud Biobank (18). Genomic DNA isolation from peripheral blood was performed according to standard procedures.

### Genotyping

Genotyping data based on whole exome sequencing (19), single-molecule molecular inversion probes in combination with next generation sequencing (20), or a customized HumanCoreExome array (2) was available for the individuals included in this study. All genotyping passed quality control as described in the original studies (2, 19, 20). We filtered for carriers of rare (minor allele frequency <1%) variants in *CFI* with a CADD score >20, and normal protein secretion based on *in vitro* testing (8). All identified *CFI* variants were confirmed with Sanger sequencing, unless they were identified in more than one genotyping platform. *CFI* variants are annotated based on NM_000204.4 and the GRCh37 (hg19) build of the human genome, and the protein numbering annotation includes the signal peptide. CADD scores of the identified *CFI* variants were determined with the annotation pipeline of the Human Genetics Department of the Radboud university medical center.

### Recombinant Expression and Purification of Mutant and Wildtype FI

Based on our previous report on the effect of rare coding variants in *CFI* on expression (8), 11 variants with a predicted damaging effect (CADD>20), but with no effect on FI secretion levels, were selected for protein purification (**Supplementary Table 1**). Full-length *CFI* was cloned with a His-tag between the signal sequence and the sequence encoding for mature FI in a pcDNA3 vector (21), and variants were introduced by site-directed mutagenesis and wildtype (WT) and mutant FI proteins were expressed as described previously (8). HEK293T cells were maintained in Dulbecco’s Modified Eagle Medium (DMEM, Gibco, USA) supplemented with 10% fetal calf serum (Sigma, USA), 1 mM sodium pyruvate (Sigma), 100 units/ml penicillin and 0.1 mg/ml streptomycin (Sigma). Cells at approximately 90% confluency were transfected with constructs containing WT or mutant FI using lipofectamine 2000 (Invitrogen, USA) according to manufacturer’s instructions. After recovery in supplemented DMEM, transfected cells were maintained for two days in serum-free OptiMEM Glutamax (Gibco, USA) before conditioned media were collected. Cell debris was removed from supernatants by centrifugation. Secreted FI was purified with HisPur Ni-NTA spin columns (ThermoFisher Scientific, USA) according to manufacturer’s instructions. The entire purification was performed at 4*°*C or on ice.

### SDS-PAGE and Silver Staining

For analysis of the purified FI protein, samples were diluted 2x in Tris-buffered saline (TBS, 50mM trisHCl, 150mM NaCl, pH7.4), denatured with NuPAGE lithium dodecyl sulfate ((LDS) sample buffer (ThermoFisher Scientific, USA) under non-reducing conditions, and separated on NuPAGE 4-12% Bis-Tris gel (Invitrogen, USA). The NuPAGE were fixed (50% methanol, 12% acetic acid, 0.0185% formaldehyde), washed with 50% ethanol and sensitized (0.8 mM Na_2_S_2_O_3_·5H_2_O). After rinsing with water, the gels were stained (11.8 mM AgN0_3_, 0.028% formaldehyde), rinsed again and then treated with developing solution (0.57M Na_2_CO_3_, 0.02 mM Na_2_S_2_O_3_·5H_2_O, 0.0185% formaldehyde). Developing was stopped by incubation with fixation solution prior to taking pictures.

### SDS-PAGE and Western Blot

To determine the processing of the variants located close to the FI linker region, conditioned supernatants of HEK293T cells transfected with *CFI* were denatured under reducing conditions with NuPAGE LDS sample buffer supplemented with 50mM Dithiothreitol. After separation on NuPAGE 4-12% Bis-Tris gels, protein was transferred to a polyvinylidene difluoride membrane. After transfer, the membrane was treated with superblock (ThermoFisher Scientific, USA) and then incubated with 500x diluted goat anti-human FI (Quidel, USA). For detection, the membrane was incubated with Donkey anti-goat IRDye800 (Rocklands, USA) antibody diluted 10,000x. For imaging the Odyssey infrared system was used (Li-Cor, USA).

### FI, FH, and C3bBbP ELISA

Sandwich enzyme-linked immunosorbent assay (ELISA) was used to determine FI, FH, and C3bBbP concentrations in EDTA plasma and FI concentrations after purification of recombinantly expressed FI. High binding microplates (Greiner, Austria) were coated with 1,000x diluted sheep anti-human FI antibody (LabNed, Netherlands), with 10,000x diluted goat anti-human FH (Quidel, USA), or with 1,000x diluted mouse anti-human Factor P#2 (Quidel, USA) in coating buffer (15 mM Na_2_CO3*10 H_2_O, 35 mM NaHCO_3_, pH 9.6). Anti-FI coated plates were blocked first with SuperBlock (ThermoFisher Scientific, USA) and then with 1% bovine serum albumin (BSA, Sigma, USA), anti-FH and anti-C3bBbP coated plates were blocked with 1% BSA (Sigma, USA). The plates were washed four times with 200 μl phosphate buffered saline (PBS) with 0.02% Tween (Merck, Germany) after blocking and between all the following incubation steps. EDTA plasma samples for the FI ELISA and FH ELISA were diluted 750x and 5,000x in PBS with 0.02% Tween and 0.2% BSA. Serum-purified FI (CompTech, USA) and serum-purified FH (CompTech, USA) were used as standards, and the samples were incubated 2h at room temperature and 1h at 37°C, respectively. EDTA plasma samples for the C3bBbP ELISA were diluted 10x in PBS-Tween 10 mM EDTA and incubated for 1h at 4°C. As a standard, the international complement standard #2 was used (22). After applying the samples, FI was detected with 1 μg/ml mouse anti-human FI (OX-21, ProSci, USA) incubated for 2h at room temperature. FH and its alternative splice product FHL-1 were detected with 10,000x diluted mouse anti-human FH (Abcam, UK) incubated for 1h at 37°C, and C3bBbP complexes were detected with 2,000x diluted rabbit anti-human C3c (Siemens, Germany) incubated for 1h at room temperature. The secondary antibodies were horseradish peroxidase labeled IgGs and were incubated for 1h at room temperature to detect FI and C3bBbP, and incubated 1h at 37°C to detect FH. For FI and FH, goat anti-mouse (Dako, Germany) was diluted 2,500x and 2,000x, respectively, and for C3bBbP 2,000x diluted goat anti-rabbit (Dako, Germany) was used. Detection was done with o-phenylenediamine dihydrochloride substrate (Dako, Germany), diluted according to manufacturer’s instructions. Each sample was measured in duplicate.

### C3b Degradation and iC3b ELISA

The ability of FI either in serum of *CFI* rare variant carriers and non-carriers or with purified recombinantly expressed mutant protein to degrade C3b was determined. Serum samples were diluted 150x in Tris-buffered saline (TBS). Purified FI protein concentrations were normalized against purified WT FI based on the absorbance measured with FI ELISA, and diluted 4x in TBS. A final concentration of 15 μg/ml C3b (CompTech, USA) and 3 µg/ml FH (CompTech, USA) was added to each sample. The reaction mixture was incubated for 10 min at 37*°*C for serum samples, and for 3h at 37°C for purified FI. A shorter incubation time was used for serum samples, in order to prevent that degradation by the FI WT allele masks the effect of the mutant allele. In addition, to determine the specificity of the iC3b ELISA, serum samples of three randomly selected individuals from the EUGENDA cohort were incubated with the FH/C3b reaction mix, C3b only, or with TBS for 90min at 37°C (**Supplementary Figure 2A**). Then, the samples were immediately diluted 40x in PBS with 0.02% Tween and applied to high binding microplates (Greiner, Austria) coated with mouse-anti iC3b (A209, Quidel, USA) 2,500x diluted in coating buffer (15 mM Na_2_CO_3_*10 H_2_O, 35 mM NaHCO_3_, pH 9.6) and blocked with 1% BSA (Sigma, USA). All following incubation steps were performed for 1h at room temperature. iC3b (CompTech, USA) was used as a standard. The plates were washed four times with 200 μl PBS 0.02% Tween after coating, after blocking and between each of the following steps. Captured iC3b was detected with 2,000x diluted rabbit anti-human C3c (Siemens, Germany), followed by 2,000x diluted goat anti-rabbit (Dako, Germany). Color was developed with o-phenylenediamine dihydrochloride substrate (Dako, Germany), diluted according to manufacturer’s instructions. Each sample was measured in duplicate. The level of degradation was confirmed with silver staining in a fraction of the degradation mix with recombinantly expressed FI (**Supplementary Figure 2B**).

### Protein Structure Analysis

Amino acids affected by the selected *CFI* variants were visualized on the protein structure of the FI-mini-FH-C3b complex [protein data bank, PDB 5O32 (23)] using YASARA version 13.9.8 (24).

### Statistical Analyses

Statistical analyses were performed with IBM SPSS Statistics for Windows, Version 25.0 (BM Corp. Released 2017. Armonk, NY: IBM Corp.) and GraphPad Prism version 9.1.2 for Windows (GraphPad Software, San Diego, California USA). The normal range of FI, FH, and C3bBbP plasma levels and the ability to generate iC3b was determined in non-carrier samples bases on the mean ± 2 SD. One-way ANOVA followed by Dunnet’s multiple comparison test was performed to compare functionality of recombinant mutant FI with WT FI.

## Results

### Cohort Description

Carriers of a rare coding variant in *CFI*, that is predicted to be pathogenic (CADD >20), were selected from the EUGENDA database. Plasma and serum samples collected on the same date were available for seven rare variant carriers, while for one carrier only plasma was available (**Table 1**). As a control for reduced function, two carriers of the c.355G>A (p.Gly119Arg) variant, previously reported to affect FI plasma levels (8, 11), were included. In addition, 14 plasma samples and 17 serum samples of age-matched non-carriers were included as controls. Of note, none of the non-carriers presented with AMD at the time of the study, but due to the relatively young age of some of the non-carriers (median 60.2 years, age range 46.3 to 72.9 years) we cannot exclude that some individuals may develop AMD later in life.

Five out of nine (56%) of the rare variant carriers reported a family history of AMD. The two unrelated carriers of the c.148C>G (p.Pro50Ala) variant presented with intermediate AMD, of which one progressed to an advanced stage during follow-up (de Breuk *et al.*, in preparation). The carriers of the variant c.1016G>A (p.Arg339Gln) are siblings and were diagnosed with no AMD and early AMD at the age of 56 and 54 years, respectively. The father also carried the p.Arg339Gln variant and presented with advanced AMD, but no plasma or serum samples were available for this study. One of the three unrelated carriers of the c.1709G>C (p.Ser570Thr) variant reported a positive family history of AMD. The carriers presented with neovascular AMD, early AMD and no AMD, respectively. It should be noted that for the individual without AMD the examination was performed at the age of 49 years, and AMD development at an older age cannot be excluded.

### Systemic Complement Levels in Carriers of Rare CFI Variants

**Figure 2:**
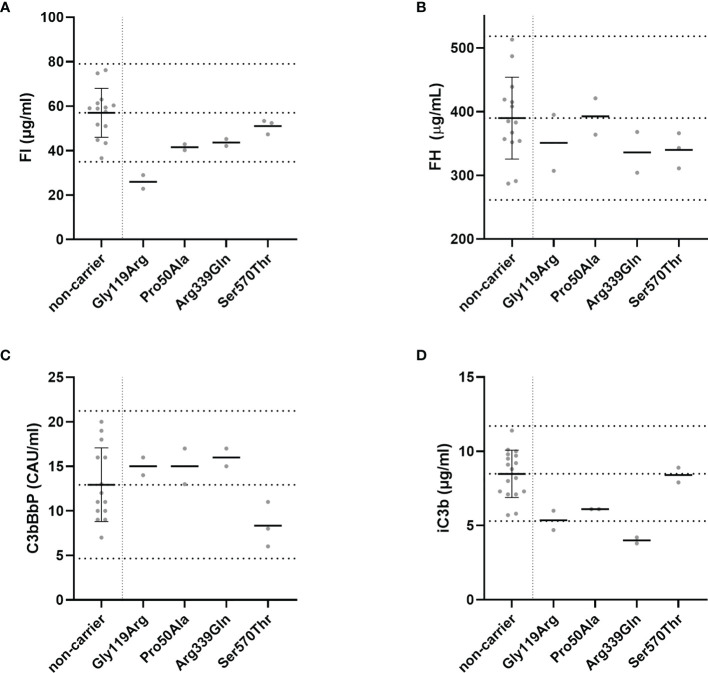
Systemic complement levels in *CFI* rare variant carriers. **(A)** FI, **(B)** FH, and **(C)** C3bBbP plasma concentrations and **(D)** the amount of iC3b generated in the C3b degradation assay were determined with ELISA. The C3b degradation was performed three times, and iC3b levels were determined in duplicate. Dotted lines indicate the mean concentrations ± 2SD determined in non-carriers. The mean ± 2SD is **(A)** 57 µg/ml ± 11 µg/ml for FI, **(B)** 390 µg/ml ± 64 µg/ml for FH, **(C)** 12.9 CAU/ml ± 4.1 CAU/ml for C3bBbP, and **(D)** 8.5 µg/ml ± 1.6 µg/ml for generated iC3b. Solid lines indicate the mean concentration for each group.

FI, FH, and C3bBbP levels were determined in each plasma sample in duplicate, and the mean for each individual is shown in **Figure 2**. Carriers of the p.Gly119Arg variant showed FI plasma levels below the normal range, and normal FH plasma levels as expected (**Figures 2A, B**). C3bBbP plasma levels of all rare variant carriers were within the normal range, but carriers of the variants p.Gly119Arg, p.Pro50Ala, and p.Arg339Gln showed C3bBbP levels above the non-carrier mean (12.93 CAU/ml), while carriers of the p.Ser570Thr variant showed C3bBbP levels lower than the non-carrier mean.

To determine the functionality of FI in serum of variant carriers, the C3b degradation assay was performed and the amount of iC3b generated as a result of FI activity was measured with ELISA. Carriers of the variant p.Arg339Gln showed iC3b generation below the normal range (**Figure 2D**). Carriers of the p.Gly119Arg and p.Pro50Ala variant generated iC3b within in the lowest quarter of the normal range, while carriers of the p.Ser570Thr variant showed iC3b levels comparable to the non-carriers mean.

### Recombinant FI Mutants Show Impaired Degradation of C3b

**Figure 3:**
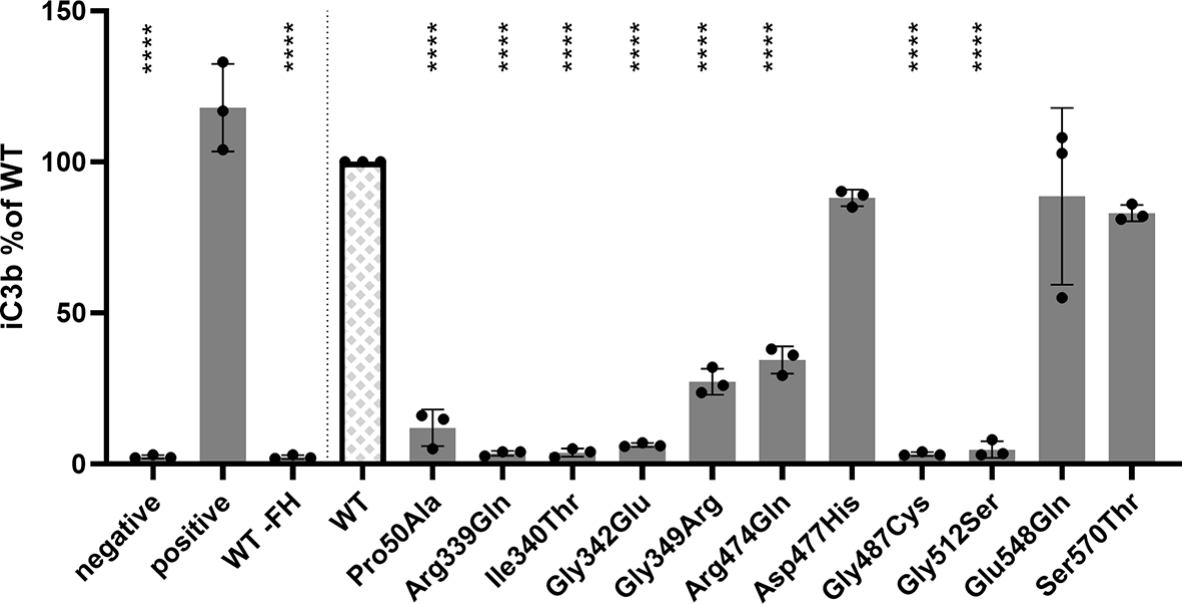
*CFI* variants affect C3b degradation. C3b degradation was performed and generated iC3b was measured with ELISA. 15 µg/ml C3b and 3 µg/ml FH were added to purified FI, and incubated for 3h at 37°C. Th negative control does not contain purified FI, and for the positive control 10 ug/ml serum purified FI was incubated with the C3b/FH mix. As additional control, recombinant WT FI was incubated with 15 µg/ml C3, but FH omitted (WT -FH). The amount of iC3b generated by the variants was normalized to the concentration generated by recombinantly expressed WT FI. Bars and error bars show the mean and SD, individual dots represent the individual measurements. Samples were compared to WT using one-way ANOVA followed by Dunnet’s multiple comparison test. P-values are indicated as ****<0.001.

Since serum and plasma samples were not available for all selected variants, and measurements in donor samples can be influenced by natural variation in concentration of complement proteins and by the presence of the WT *CFI* allele in heterozygous carriers, mutant proteins for 11 *CFI* variants were recombinantly expressed and purified to determine the ability of the *CFI* variants to degrade C3b *in vitro* (**Supplementary Figure 1**). Mutant proteins containing eight of the variants (p.Pro50Ala, p.Arg339Gln, c.1019T>C (p.Ile340Thr), c.1025G>A (p.Gly342Glu), c.1045G>A (p.Gly349Arg), c.1421G>A (Arg474Gln), c.1459G>T (Gly487Cys), and c.1534G>A (p.Gly512Ser)) showed significantly reduced generation of iC3b compared to recombinant WT FI (**Figure 3**). These findings were confirmed with silver staining (**Supplementary Figure 2**). No significant difference in iC3b generation was observed for mutant FI proteins containing the variants c.1429G>C (p.Asp477His), c.1642G>C (p.Glu548Gln), p.Ser570Thr compared to WT FI.

### Structural Protein Analysis and Impaired FI Processing for the Arg339Gln Variant

**Figure 4:**
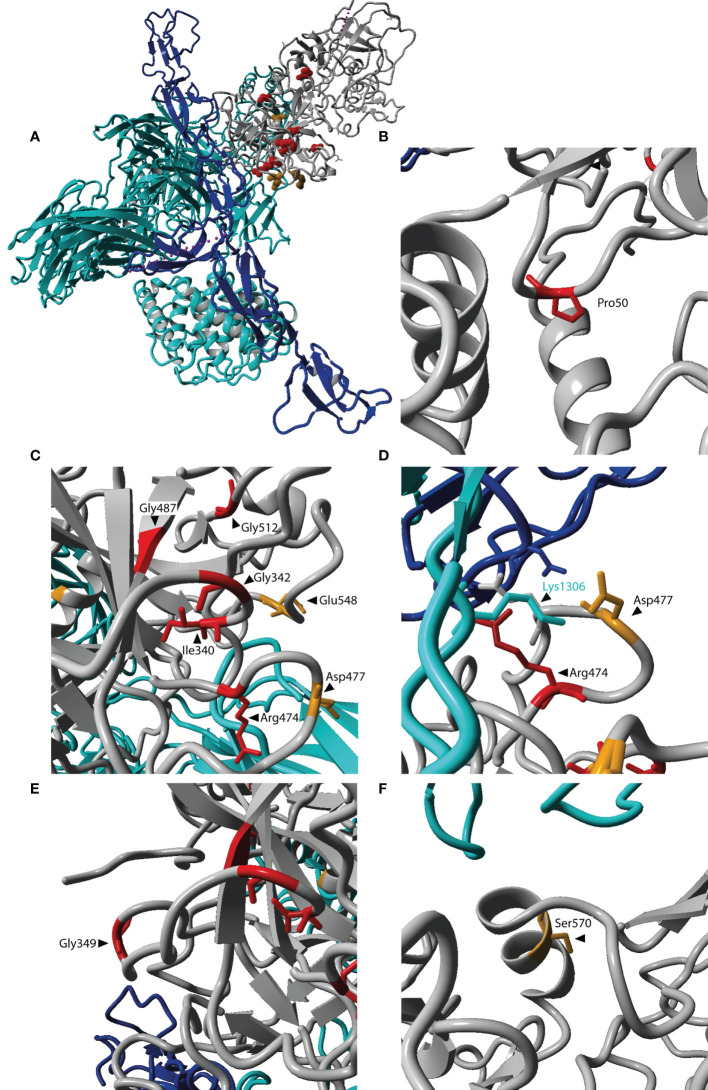
Localization of affected residues in the FI protein structure. **(A)** The three-dimensional protein structure of FI (grey) is shown in complex with C3b (cyan) and mini-FH (an artificial protein consisting of FH domains 1-4, 19-20, blue). Locations for amino acid changes included in this study are indicated in red if reduced C3b degradation was observed, and orange if no altered C3b degradation was observed. **(A)** Depicts the entire complex of FI, mini-FH and C3b. **(B–F)** are zoomed in on the affected residues. The location p.Arg339 is not included here, since it is since it is part of the cleavage site not present in the mature protein. Residues in FI are labeled in black. The residue Lys1306 located in the scissile loop of C3b is labeled in cyan. The figure was generated based on PDB 5O32 (23) in Yasara Version 13.9.8 (24).

Amino acids affected by *CFI* variants were visualized on the three-dimensional protein structure of the FI-miniFH-C3b complex (**Figure 4A**). In this structure FI is shown in complex with C3b and an artificial FH construct (miniFH) consisting of FH domains 1-4 and 19-20, which are important for C3b interaction. The p.Pro50Ala variant is located in the FI membrane attack complex domain (FIMAC) domain (**Figures 1** and **4B**), while nine other variants are located in the SP-domain (**Figures 1** and **4C–F**). The residues Pro50, Gly342, Gly487, Gly349, Gly512, and Ser570 are not directly located in the interface with mini-FH or C3b (**Figures 4B, C, E, F**), while the residues Ile340, Arg474, Asp477, and Glu548 are located on the interface with C3b (**Figures 4C, D**). The variant p.Arg339Gln is not shown, since it is part of the FI linker region containing the residues 336-339 (Arg, Arg, Lys, Arg) which are cleaved during posttranslational processing of FI. Western blot analysis of the variants located in or near the linker region (p.Arg339Gln, p.Ile340Thr, and p.Gly342Glu) was performed to determine whether FI is processed comparable to WT. HEK293 cells overexpressing WT FI, secrete both processed and unprocessed FI (21), resulting in three visible bands under reducing conditions: full-length (unprocessed) FI, the heavy chain, and the light chain of FI (**Supplementary Figure 3**). The variants c.979T>C (p.Cys327Arg), c.982G>A (p.Gly328Arg), c.1015C>T (p.Arg339*) significantly impair FI secretion, as described before (8). For the variants p.Ile340Thr and p.Gly342Glu the heavy and light chain of FI are visible, indicating processing to mature FI. For the variant p.Arg339Gln, however, only the unprocessed full-length FI band is visible, indicating that this variant affects processing of FI into the heavy and light chains.

## Discussion

In this study we performed functional analysis of rare coding variants in *CFI* that are predicted to be damaging, but do not significantly affect FI secretion levels (8). We determined FI, FH, and C3bBbP plasma levels in carriers of the variants p.Pro50Ala, p.Arg339Gln and p.Ser570Thr, and also tested the ability of serum FI to degrade C3b *in vitro* (**Figure 2**). Furthermore, we tested the ability of mutant FI proteins containing 11 different amino acid changes to degrade C3b, of which eight variants cause impaired FI function. (**Figure 3**).

Consistent with our previous data, FI levels in these carriers were within the control range, except for carriers of the p.Gly119Arg variant, which is known to decrease FI levels (8, 9, 11). None of the here included individuals carried rare genetic alterations in the *CFH* gene, and FH levels were consistently within the control range for all *CFI* variant carriers. In carriers of the p.Pro50Ala variant both reduced and normal FI plasma levels have been reported previously (10, 12, 13, 25–28). In the current study we observe FI plasma levels in the normal range for two carriers (**Figure 2A**).

The p.Pro50Ala variant has been identified in both AMD and aHUS patients (**Supplementary Table 1**), and is located in the FIMAC domain (**Figures 1**, **4B**). This domain binds to the C-terminal C345c (CTC) domain of C3b (23), and was shown to be essential for the ability of FI to degrade C3b and C4b (29). In line with this, the recombinantly expressed mutant protein containing the p.Pro50Ala variant showed significantly reduced C3b degradation compared to WT FI (**Figure 3**). Furthermore, in donor samples, both carriers showed C3bBbP convertase plasma levels that were above the control mean, and levels of the degradation product iC3b were below the control mean (**Figures 2C, D**). Although still within the control range, these differences agree with the data obtained with recombinant FI analysis. In line with our findings, a mild effect on degradation of C3b in fluid phase, and reduced C3b degradation on sheep erythrocytes has been previously observed with recombinant protein carrying the p.Pro50Ala substitution (28). Altogether, the data from our and previous findings indicate that the p.Pro50Ala variant is likely pathogenic.

To date, the variant p.Arg339Gln has been reported only in AMD patients. This variant affects the Arg339 located in the linker region of FI (**Figure 1**), which is cleaved during posttranslational processing to mature, functional FI (30, 31). Unprocessed FI does not cleave C3b (31), and for recombinant protein with the p.Arg339Gln substitution only unprocessed FI is visible (**Supplementary Figure 3**). In line with this observation, this variant led to reduced iC3b generation both in plasma of carriers (**Figure 2**) and with recombinantly expressed protein (**Figure 3**). In addition, carriers of this variant presented with C3bBbP plasma levels above the control mean. In agreement with our findings, a single carrier of this variant with normal FI plasma levels, but reduced iC3b generation with the C3b degradation assay in serum has previously been reported (13). Taken together, this variant impairs posttranslational processing of FI, resulting in impaired function. This indicates a pathogenic effect of the p.Arg339Gln variant.

The remaining nine variants tested are all located in the SP-domain of FI (**Figure 1**). The residue Ile340 stabilizes the oxyanion hole of FI, supporting the proteolytic function of FI (23). In line with this a loss of function in C3b degradation was observed here and previously for the variant p.Ile340Thr (32, 33). This variant has been identified in both AMD and aHUS patients, and is classified as pathogenic.

The variant p.Arg474Gln has been identified in both AMD and aHUS patients. It affects an arginine residue at the interface with the Thioester-containing domain (TED) of C3b, specifically the scissile loop consisting of the C3b residues 1300-1306 (**Figures 4C, D**) (23), and is part of a short loop of FI (residue 471 to 485), which is in contact with mini-FH (23). This variant results in a significant reduction of iC3b generation with recombinant protein (**Figure 3**). No data on the ability to degrade C3b has been previously reported for the p.Arg474Gln variant. But in a single carrier with the additional variant c.772G>A, causing skipping of exon 5 and known to cause FI deficiency, no alternative pathway function was detected. This observation is likely due to extensive complement activation and secondary consumption of its components (34). The contribution of p.Arg474Gln variant in this case was unclear. Based on our findings we can now classify the p.Arg474Gln variant as likely pathogenic on its own.

The variant p.Gly487Cys has been identified in AMD patients (**Supplementary Table 1**). Even though this variant is not located on the binding interface of the FI SP-domain with C3b (**Figure 4C**), a significantly reduced generation of iC3b by the mutant recombinant protein was observed (**Figure 3**). In line with our findings, reduced generation of iC3b has been previously reported in a single, heterozygous carrier (13), suggesting this variant is likely pathogenic.

For the remaining six variants no functional analysis has been previously reported. The mutant recombinant proteins containing three variants p.Gly342Glu, p.Gly349Arg, and p.Gly512Ser show significantly reduced iC3b generation (**Figure 3**), indicating that these variants are pathogenic. The recombinant variants c.1429G>C (p.Asp477His), c.1642G>C (p.Glu548Gln) and p.Ser570Thr exhibited normal function compared to WT protein (**Figure 3**), and are likely benign. Moreover, carriers of the p.Ser570Thr variant have a mean C3bBbP plasma level of 8.3 CAU/ml, which is below the control mean of 12.93 CAU/ml, and show comparable levels of iC3b generation to the controls. These findings further support a benign nature of the p.Ser570Thr variant.

Here we show that eight (73%) out of 11 variants predicted as damaging based on CADD score cause significantly reduced C3b degradation compared to WT. For nine variants (p.Arg339Gln, p.Gly342Glu, p.Gly349Arg, p.Arg474Gln, p.Asp477His, p.Gly487Cys, p.Gly512Ser, p.Glu548Gln, and p.Ser570Thr) the conclusive pathogenicity analysis with recombinant protein was performed here for the first time. In our previous work, we have shown that the CADD score >20 is a useful predictor of lower FI expression (8). In this study, we have shown that variants that do not alter FI protein expression, but have a CADD score above 20 are likely to affect FI function. In future experiments the predictive power of a CADD score <20 for benign variants should be addressed.

Furthermore, this study indicates that C3b degradation together with the monitoring of iC3b formation *in vitro* by ELISA is a useful tool to assess functional pathogenicity of *CFI* missense variants both in serum samples of carriers and with FI recombinant protein.

Future functional study with a larger number of variants with various CADD scores is needed to further investigate utility of CADD score and iC3b degradation assay as a screening tool for FI variants.

In summary, here we report eight rare coding variants in *CFI* affecting the ability of FI to degrade C3b. For three variants normal ability to degrade C3b was observed, indicating they are likely benign. But this needs to be confirmed in further functional studies including other co-factors. The here reported data will improve the interpretation of rare variants observed in AMD and aHUS patients, and thereby aid personalized treatment options.

## Data Availability Statement

The original contributions presented in the study are included in the article/**Supplementary Material**. Further inquiries can be directed to the corresponding author.

## Ethics Statement

The studies involving human participants were reviewed and approved by Local ethics committee region Arnhem/Nijmegen on Research Involving Human Subjects. The patients/participants provided their written informed consent to participate in this study. Written informed consent was obtained from the individual(s) for the publication of any potentially identifiable images or data included in this article.

## Author Contributions

Study design: SJ, SK, SN, AMB, LH, AH, and EV. Provision of patient information and material: AdB, BB, and CH. Experimental procedures: SJ. Data analysis and interpretation: SJ and EV. Manuscript: SJ and EV. All authors contributed to the article and approved the submitted version.

## Funding

This work was supported by a collaborative research agreement with Gemini Therapeutics, Inc.

## Conflict of Interest

Author SK was employed by company Gemini Therapeutics Inc.AH is a consultant for Ionis Pharmaceuticals, Gyroscope Therapeutics, Gemini Therapeutics and F. Hoffmann-La Roche.

The remaining authors declare that the research was conducted in the absence of any commercial or financial relationships that could be construed as a potential conflict of interest.

This study received funding from Gemini Therapeutics Inc. The funder’s employer (SK) had contributed to study design and preparation of the manuscript.

## Publisher’s Note

All claims expressed in this article are solely those of the authors and do not necessarily represent those of their affiliated organizations, or those of the publisher, the editors and the reviewers. Any product that may be evaluated in this article, or claim that may be made by its manufacturer, is not guaranteed or endorsed by the publisher.

## Supplementary Material

Supplementary Figure 1 Purified recombinant FI variants. Purified recombinant FI was diluted 2x, denatured under non-reducing conditions and separated by SDS-PAGE and visualized by silver staining. Equal volumes were loaded to visualize the yields after purification. The purification efficacy was not optimized for maximum yield for each variant. SP FI, serum purified FI (CompTech, USA); WT, wildtype recombinant FI.

Supplementary Figure 2 Confirming the specificity of the iC3b ELISA. **(A)** Three randomly selected serum samples from the EUGENDA database were diluted 150x in TBS and incubated for 90min at 37°C either without further addition of C3b (serum), with the addition of 15 µg/ml C3b (serum + C3b), or with the addition of 15 µg/ml C3b and 3 µg/ml FH (serum + C3b + FH). The positive control contains 10 µg/ml serum purified FI and 15 µg/ml C3b and 3 µg/ml FH, while in the negative control FI was omitted. Black bars represent the mean for each group. The C3b degradation was performed once, and iC3b was determined in duplicate in each sample. **(B)** The C3b degradation was performed with recombinantly expressed and purified mutant and WT FI. The sample was used for measurement with the iC3b ELISA (**Figure 3**), and the remaining volume was denatured under reducing conditions and proteins separated on SDS page and visualized with silver staining.

Supplementary Figure 3 The variant c.1016G>A (p.Arg339Gln) affects FI processing. Conditioned supernatants of HEK293T cells transfected with *CFI* constructs carrying variants affecting residues close the to the linker region were separated with SDS-PAGE under reducing conditions, and FI was visualized with western blot. The variants c.979T>C (p.Cys327Arg), c.982G>A (p.Gly328Arg), c.1015C>T (p.Arg339*) affect FI secretion levels (8). WT FI, c.1019C>T (p.Ile340Thr), and c.1025G>A (p.Gly342Glu) show partial processing, while for the variant c.1016G>A (p.Arg339Gln) only one band of unprocessed FI is visible. Mock, empty vector transfected control; WT, wildtype FI.

## Abbreviations

aHUS, atypical hemolytic uremic syndrome; AMD, age-related macular degeneration; BSA, bovine serum albumin; CADD, Combined Annotation Dependent Depletion; CFI, complement factor I; FB, Factor B; FH, Factor H; FI, Factor I; FP, Properdin; PBS, phosphate buffered saline; TBS, tris-buffered saline; WT, wildtype; TED, Thioester containing domain.
