# Supplementary figures and images for: Functional Analysis of Variants in Complement Factor I Identified in Age-Related Macular Degeneration and Atypical Hemolytic Uremic Syndrome
Source: Front Immunol. 2022 Jan 5;12:789897. doi: 10.3389/fimmu.2021.789897 (PMC8766660; doi:10.3389/fimmu.2021.789897)

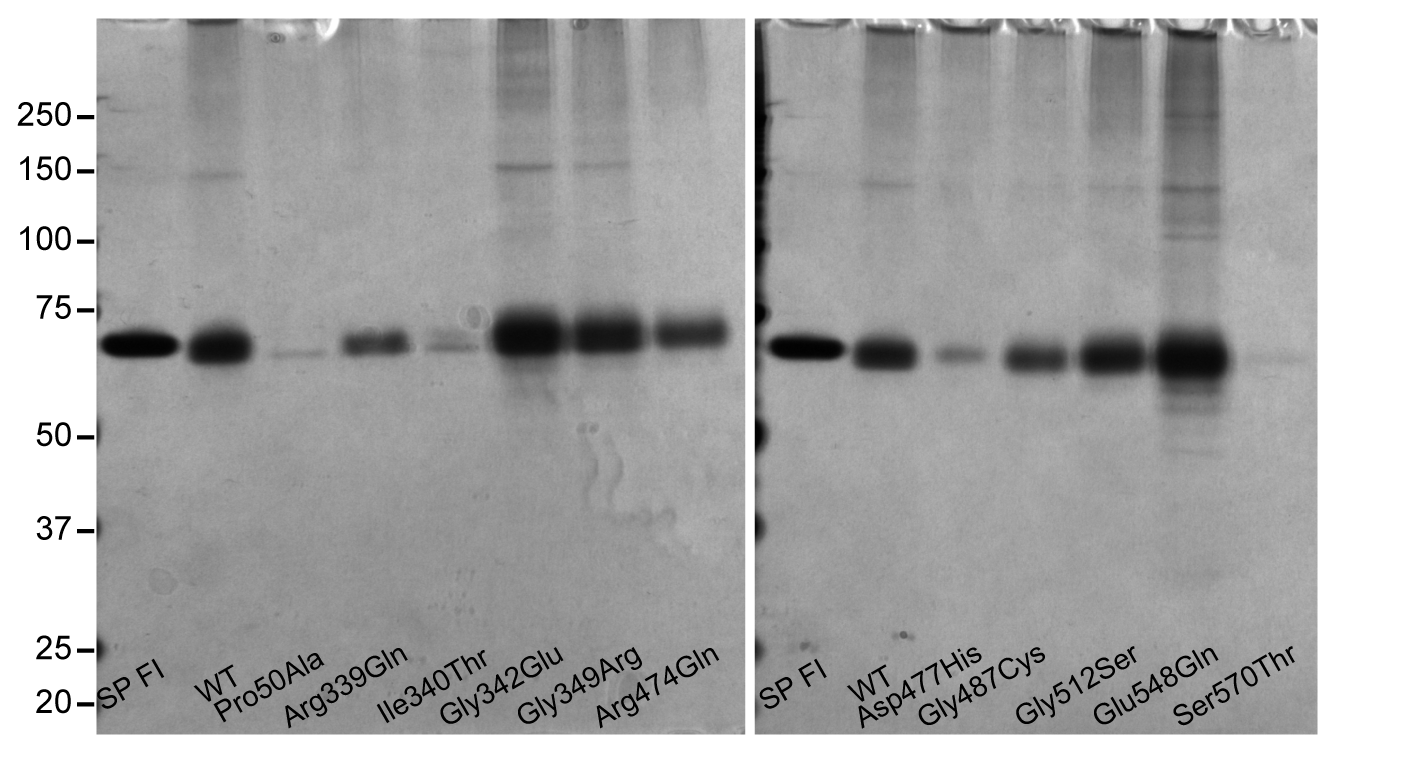

Supplement: Supplementary Figure 1 — Purified recombinant FI variants. Purified recombinant FI was diluted 2x, denatured under non-reducing conditions and separated by SDS-PAGE and visualized by silver staining. Equal volumes were loaded to visualize the yields after purification. The purification efficacy was not optimized for maximum yield for each variant. SP FI, serum purified FI (CompTech, USA); WT, wildtype recombinant FI. [file Image_1.tif]

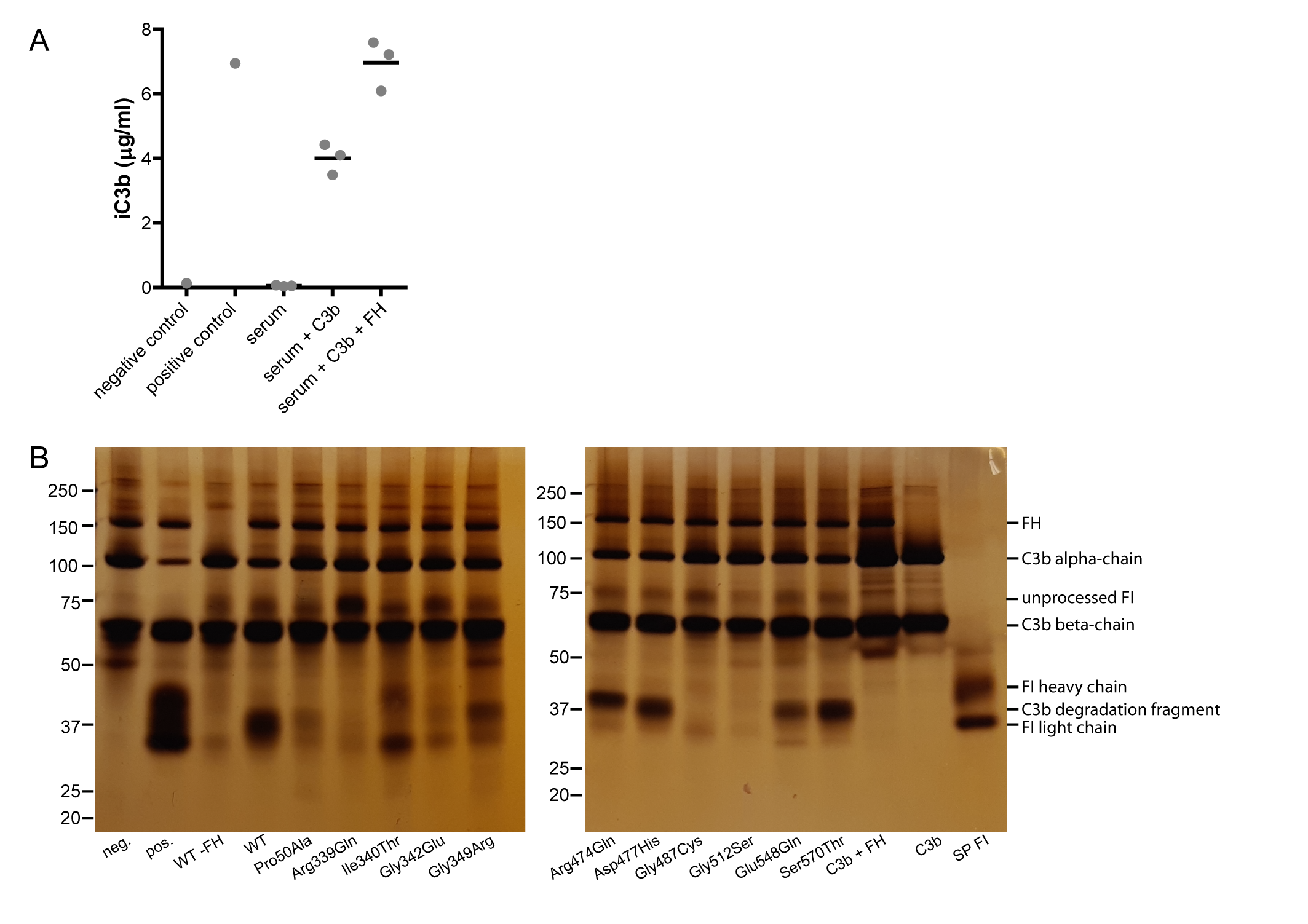

Supplement: Supplementary Figure 2 — Confirming the specificity of the iC3b ELISA. (A) Three randomly selected serum samples from the EUGENDA database were diluted 150x in TBS and incubated for 90min at 37°C either without further addition of C3b (serum), with the addition of 15 µg/ml C3b (serum + C3b), or with the addition of 15 µg/ml C3b and 3 µg/ml FH (serum + C3b + FH). The positive control contains 10 µg/ml serum purified FI and 15 µg/ml C3b and 3 µg/ml FH, while in the negative control FI was omitted. Black bars represent the mean for each group. The C3b degradation was performed once, and iC3b was determined in duplicate in each sample. (B) The C3b degradation was performed with recombinantly expressed and purified mutant and WT FI. The sample was used for measurement with the iC3b ELISA ( Figure 3 ), and the remaining volume was denatured under reducing conditions and proteins separated on SDS page and visualized with silver staining. [file Image_2.tif]

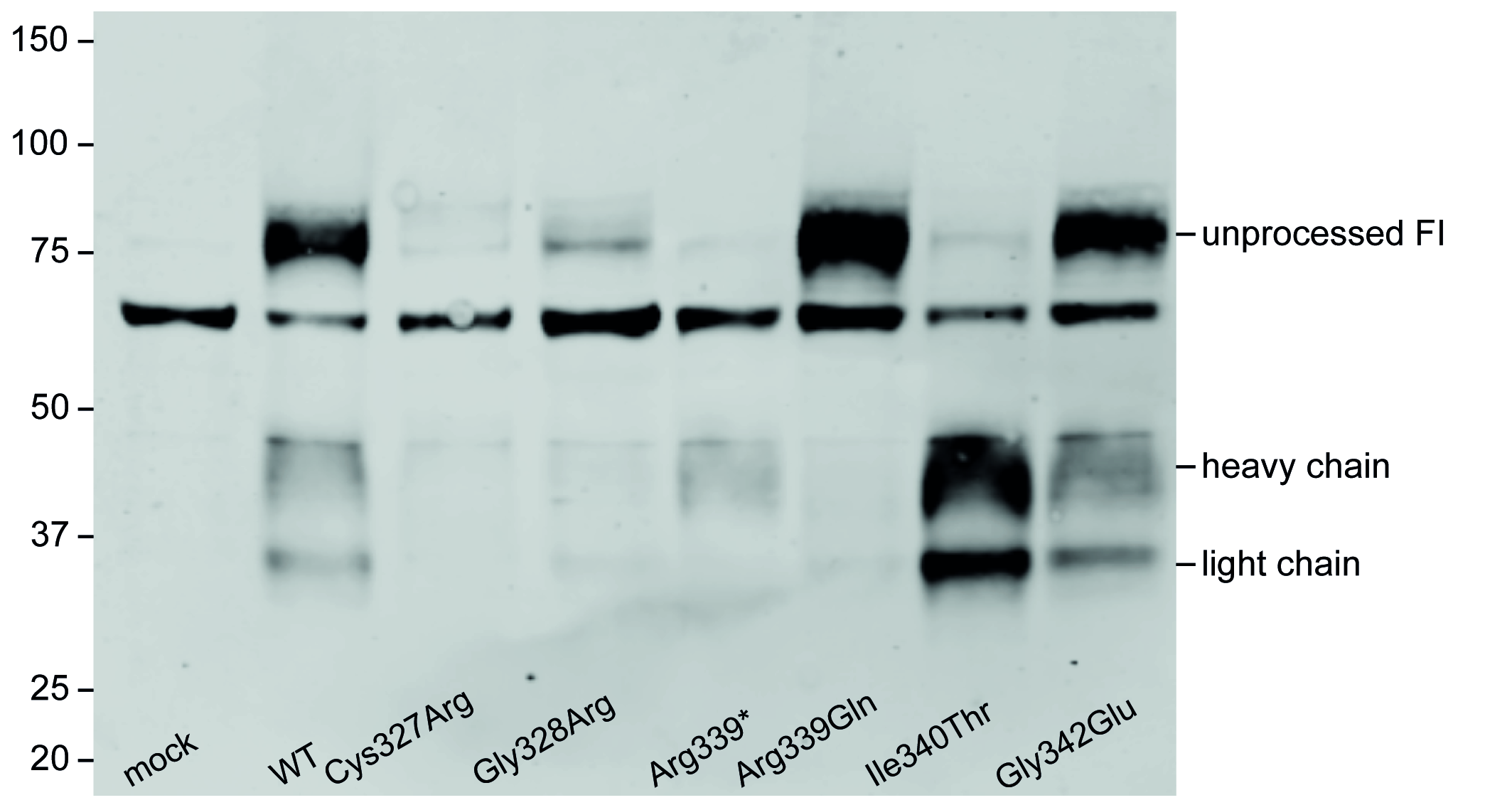

Supplement: Supplementary Figure 3 — The variant c.1016G>A (p.Arg339Gln) affects FI processing. Conditioned supernatants of HEK293T cells transfected with CFI constructs carrying variants affecting residues close the to the linker region were separated with SDS-PAGE under reducing conditions, and FI was visualized with western blot. The variants c.979T>C (p.Cys327Arg), c.982G>A (p.Gly328Arg), c.1015C>T (p.Arg339*) affect FI secretion levels (8). WT FI, c.1019C>T (p.Ile340Thr), and c.1025G>A (p.Gly342Glu) show partial processing, while for the variant c.1016G>A (p.Arg339Gln) only one band of unprocessed FI is visible. Mock, empty vector transfected control; WT, wildtype FI. [file Image_3.tif]
